# Alternating between active and passive facilitator roles in simulated scenarios: a qualitative study of nursing students’ perceptions

**DOI:** 10.1186/s41077-022-00233-0

**Published:** 2022-10-29

**Authors:** Hilde Solli, Thor Arne Haukedal, Sissel Iren Eikeland Husebø, Inger Åse Reierson

**Affiliations:** 1 Department of Nursing and Health Sciences, Faculty of Health and Social Sciences, Clinical Competence in Nursing Education Research Group, University of South-Eastern Norway, Raveien 215, 3184 Borre, Vestfold and Telemark Norway; 2 Department of Quality and Health Technology, Faculty of Health Sciences, University of Stavanger, Post Box 8600, 4036 Stavanger, Rogaland Norway

**Keywords:** Active or passive facilitating, Facilitating, Facilitator, Facilitator role, Nursing students, Nursing students’ perspectives, Simulation, Simulation-based learning, Structured text condensation

## Abstract

**Background:**

High-fidelity simulation refers to realistic interactivity between students and an advanced simulator. During simulated scenarios, the facilitator often needs to provide guidance to the active students to bridge the gap between their insufficient practical nursing skills and clinical learning needs. Facilitators’ guidance should support students in problem-solving and help them progress in their simulation experiences. The aim of this study was to explore and describe nursing students´ perspectives on the facilitator’s role during simulated scenarios.

**Methods:**

A qualitative design was used. Thirty-two nursing students participated in five focus groups conducted immediately after a 2-day high-fidelity simulation course in the second year of their Bachelor of Nursing in Norway. The analysis used structured text condensation.

**Results:**

One main category, “Alternating between active and passive facilitation,” emerged along with three sub-categories: (1) practical support: the facilitator played an important role in ensuring the flow of the simulated scenarios. Some students sought cues from the facilitator or responses to their actions. Other students wanted to act independently, reassured by the possibility of asking for assistance. (2) Guiding communication: the facilitator was important to students in paving their way to achieve the learning outcomes. The way facilitators supported students influenced students’ understanding and their feelings about how they handled the situation and whether they achieved the learning outcomes. (3) Emotional influence: the facilitator’s presence in the simulation room during the simulated scenarios influenced students’ emotions, for example having a calming or aggravating effect or making them feel distressed. In some cases, students were undisturbed.

**Conclusions:**

The facilitation of simulated scenarios requires special skills in providing individually suitable cues at the right time to students with a variety of learning preferences. It is vital that facilitators have well-developed relational, pedagogical, and emotional competence combined with clinical, technical, and simulation-based learning skills in monitoring different learning preferences. As the facilitator role is challenging and complicated, more research is needed to explore how facilitators could monitor and adjust cues individually in simulated scenarios.

**Supplementary Information:**

The online version contains supplementary material available at 10.1186/s41077-022-00233-0.

## Background

Simulation-based learning (SBL) is an educational technique frequently used in nursing education to prepare students for their practical placement [1–3]. High-fidelity simulation (HFS) refers to realistic health care experiences based on a high level of interactivity for learners. To provide real-life situations, various simulation modalities can be used: computerized mannequins, humans, task trainers, or virtual reality [4]. Providing a realistic scenario entails “creating an environment that mimics that of the learner’s work environment; realism includes the environment, simulated patient, and activities of the educators, assessors, and/or facilitators” [4]. In a simulation laboratory, students can train in a safe environment, according to pre-defined learning outcomes in line with clinical learning needs and clinical learning requirements [3–5]. In the simulation, a facilitator guides students through briefing, simulation scenario, and debriefing sessions to achieve learning outcomes [4, 6–8].

Facilitators need to provide guidance during simulations to bridge the gap between students’ insufficient understanding and the reality of clinical practice [8]. Simulating unforeseen occurrences is a way of acquiring such experience [9]. Facilitators should have adequate practical nursing skills, simulation experience, and pedagogical approaches to time their responses appropriately and meet students’ different needs for guidance during simulated scenarios [10]. Different types of guidance include delivering cues such as triggers, prompts, and hints via instructional support such as telephone, laboratory reports, questions, answers, and practical assistance [7, 11]. These cues should be given in a way that supports students in problem-solving and enables them to progress while participating in the simulation [1, 2, 11–13].

Sociocultural learning theory defines the zone of proximal development (ZPD) as the space between what a student might learn on their own and what they might learn with skilled support [14–17]. Facilitators should time their cues to give students the chance to problem-solve on their own before guiding them towards their ZPD [14, 17–19]. The frequency of feedback during simulation needs to be considered carefully [20]. While extensive feedback might be helpful for students, it might also promote stress and insecurity [20]. Some students might wait for cues before they take their next step, which can hinder the development of problem-solving skills [2, 13]. The cognitive load theory, however, claims that to maximize learning, tasks should be challenging, encouraging and appropriate to the learner's level of expertise. If the task is too difficult, it may negatively affect learning outcomes [21].

Despite the importance of facilitators, there are, to our knowledge, only a few studies addressing students’ perspectives on facilitators’ role during simulation scenarios. Two studies [22, 23] found that extra information from a facilitator who stayed in the room as either a helper or a bystander during the scenario helped participants advance in their problem-solving process. Some students wanted minimal assistance, while others wanted guidance during the scenario. Consequently, facilitators had to carefully consider when to switch from a bystander to a helper role. Facilitators’ guidance during the simulation scenario also sharpened students’ thinking so they approached the situation in the best possible way [24]. However, cues that interrupted problem-solving were mostly unwanted [23]. The students preferred silence, which allowed them to concentrate on self-reflection and demonstrate problem-solving [23].

The paucity of studies describing students’ preferences regarding facilitator behavior during simulation indicates the need for more research on this topic. Students’ perspectives are essential to improve simulation as a learning method. Therefore, the aim of this study was to explore and describe nursing students’ perspectives on the facilitator’s role during simulated scenarios. Hopefully, the results from the present study can be used to improve facilitators’ competence in the future.

## Methods

### Design

The study used an exploratory-descriptive design and a qualitative approach, which is recommended when a phenomenon is poorly studied [25]. Focus groups were used as a data collection method. The design allowed the interviewer to add questions to the informants’ responses to achieve the fullest description of the topic until saturation [25]. This method is well-suited to uncovering participants’ experiences, perspectives, attitudes, and views about a practice. Focus group interviews make it possible to illuminate multiple perspectives since participants might stimulate each other to participate in discussion about the topic [26]. To enhance rigor, we consolidated a checklist for reporting on qualitative research studies [27].

### Participants

A purposive sample was used as the university had two cohorts of students from the second year of a bachelor’s program in nursing taking the same simulation course [26, 28]. A total of 137 students (83 full-time students and 54 part-time students) from both cohorts were invited to participate in the study. After giving written consent, a total of 38 students (24 full-time students and 14 part-time students) were included in the study.

### Setting

The study was conducted in the simulation center at a university in south-eastern Norway. The students were informed about the study in class and via the institution’s digital learning platform. The compulsory simulation course was conducted before the students’ first clinical placement in surgical or medical units. The simulation framework involved a briefing session, a simulated scenario, and a debriefing session.

In the simulation course, students were divided into 15 groups of 7–11 participants. The students participated in a total of six scenarios over a 2-day period. The scenarios focused on patients with deteriorating health conditions (Additional file 1) [29]. The complexity of the scenarios varied; however, the order was random. The learning outcomes included assessing airway, breathing, circulation, disability, and exposure (ABCDE) [30]; prioritizing relevant nursing actions; and executing effective communication and clear leadership. Two students acted as nurses in each scenario, with one as the leading nurse; the remaining students were observers or acted as next-of-kin. To provide HFS, three advanced simulators were used.

Six faculty attended either as facilitator or operator, all well experienced in advanced simulation. The students rotated between acting as a nurse and observing from one scenario to another. Since the simulation course was conducted prior to the students’ first hospital internship, the facilitators were instructed to assess independently when to intervene to help the simulated scenarios run smoothly. During the simulated scenarios, both student observers and the facilitator were present in the simulation room.

### Data collection

The research group developed a semi structured interview guide (Additional file 2), inspired by the NLN Jeffries simulation framework [1, 10, 12]. Data collection was carried out in December 2017 (full-time students) and May 2018 (part-time students). For both cohorts, the focus groups took place immediately after their last simulation to ensure students would recall what they had experienced during the course. Thirty-two of the 38 students who consented participated in five focus groups. Only two of six participants attended the first focus group. Since the literature supports focus groups with few participants [31], this interview was included in the data analysis. Table 1 provides an overview of the focus groups and participants.

**Table 1:** Overview of the focus groups and participants

Focus group	Participants (no.)	Full-time students: (no.)	Part-time students: (no.)	Female/male (no.)	Age (range)
**1**	2	2		1/1	38–42
**2**	9	9		7/2	20–39
**3**	8	8		6/2	20–38
**4**	6		6	5/1	21–25
**5**	7		7	7/0	22–40

The focus groups were moderated by the third author and four faculty at the university, none of whom facilitated the simulation course. All were registered nurses with academic credentials (two professors, two associate professors, and one assistant professor), skilled in moderating focus groups. Two were trained SBL facilitators. The first author informed all the moderators about the setting and objectives of the course; the moderators also observed a couple of scenarios in advance.

The present study was part of a more comprehensive study exploring the role of facilitators in all parts of SBL [6]. The focus groups lasted 60–90 min and were audio recorded. The present study focusses on data describing the simulation scenario experience.

### Analysis

A professional agency transcribed the audio-recorded files verbatim. All authors contributed to the analysis process. All were registered nurses, skilled in SBL and qualitative research methods. The data were analyzed using systematic text condensation consisting of four steps: (1) total impression and identifying preliminary themes, (2) coding and sorting meaning units into potential themes, (3) condensing decontextualized meaning units into themes, and (4) synthesizing the condensates into an analytical text [32]. Table 2 shows excerpts of the analysis process.

**Table 2:** Excerpts of the analysis process

**Preliminary** **themes** **Step 1**	Experiences with active or passive assistance, perspectives on active and passive assistance, and awareness of facilitator’s presence.
**Potential theme** **Step 2**	Emotional influence
**Condensate** **Step 3**	It was beneficial to have the possibility of “time out” with the facilitator in the room. (Several students said this about two scenarios). It was advantageous to have pointers to help us solve the task, despite the stress of the situation. Not all the scenarios provided information about the possibility of “time out”, and I can understand that it is important that the facilitator is present as a safeguard during the simulation. I was more confident when the facilitator was in the room.
**Analytical text** **Step 4**	The students also felt reassured knowing the facilitator was present and willing to help them whenever they needed. They also had the possibility to request ‘time out’ in two of the scenarios, which they appreciated very much. A student expressed, “I think it is very good that they [the facilitators] are sitting there watching us…. I don´t feel performance anxiety. It was reassuring to be told that this is a learning situation whose aim is not perfection” (Focus group 5).

The first author prepared an analytical text with suggestions for main categories and sub-categories. Wording, category content, and selected quotes were discussed several times within the team [32]. Through an iterative process of reformulating the text, the authors agreed on the organization of data into a main category and sub-categories.

## Results

The analysis identified one main category and three sub-categories. The main category, “alternating between active and passive facilitator”, conveys how facilitators need special skills to provide appropriately timed individual cues to accommodate students’ learning preferences. The first sub-category, “practical support”, shows the diversity of students’ practical support preferences during simulated scenarios. The second sub-category, “guiding communication”, captures the importance of the facilitator’s verbal and nonverbal communication in guiding students to reach their learning outcomes. The third sub-category, “emotional influence”, describes students’ emotional reactions to the facilitator’s presence in the room during the simulated scenarios.

### Practical support

This sub-category shows the variety in students’ preferences for active or passive support from the facilitator. Some students wanted help, demonstrations or explanations of how to use technical equipment or perform procedures during the simulated scenarios. Other students wanted to challenge their own limits and act without interference from the facilitator.

The students were aware of the facilitator’s presence during the simulated scenario and the possibility for support if needed. They described the facilitator as available, understanding, and able to answer questions or offer practical support if they appeared unsure. Most students seemed to prefer to receive assistance quickly without asking. If something was ambiguous during the simulated scenarios, students appreciated prompt practical support from the facilitator, for instance in handling the scope or other equipment. The students experienced that the possibility of receiving assistance quickly during the actual patient situation reduced their feeling of being left to themselves.

From the students’ point of view, it was critical to quickly get practical support from the facilitator to enhance their learning outcomes and not spend a lot of time on unimportant issues:

It is very important that the facilitator has a complete overview. When you are standing there fumbling with something or other, it is a relief to move on as soon as possible, to maximize the learning outcome. (Focus group 1)

An opposite perspective was related to situations where the facilitator was restrained in their interventions and the students had to wait to receive support during the simulated scenarios. Some students found it motivating to try to handle the situation on their own before receiving support from the facilitator:

[It’s] fine to have a facilitator who is active and helps us with technical problems. But I think it was a good thing that the facilitator did not provide help too quickly because there is something about being independent and confident in your decisions. (Focus group 4)

Students expressed diverse perspectives in terms of valuing active or passive facilitation during the simulated scenarios. The timing of facilitators’ active or passive behavior seemed to affect the flow and continuity of the simulated scenarios and influenced students’ assessment of whether the involvement improved their learning.

### Guiding communication

In this sub-category, the students described the facilitator’s verbal and nonverbal guidance as important for them to reach their learning outcomes. However, their perspectives on whether the facilitator’s communication during the simulated scenarios was supportive varied.

When they found facilitators to be restrained or passive during the simulated scenarios, some students focused on nuances in the facilitator’s response that might indicate that they were on the right track. Receiving support along the way was important for students’ self-assurance as they continued their simulation:

When I was in action in a scenario, I looked at the facilitator hoping to receive assurance that what I did was the right thing to do – making sure I was not on the wrong track. I got a little nod in reply, which I appreciated a lot. (Focus group 4)

Some students shared how their ability to achieve their learning outcomes was affected when the facilitator remained passive and did not provide any assistance. They described how terrible it felt to be in a critical situation and unable to handle the patient’s health condition. These situations were described as pivotal learning experiences since they happened in a safe learning environment.

The facilitator did not intervene when I did not quite know what to do in the cardiac pulmonary resuscitation. The feeling I had of not knowing what to do in this situation, I will always remember it. If someone had been there [to help], it would have been nice; but imagine if I were standing there alone and there was a patient, and it was me, and I didn't know what to do! Therefore, it's nice to have experienced this feeling with the manikin and not with a real person. So, I think it was a relief that no one cried out "now you have to do this and that", because I will never forget that feeling of standing there and not knowing what to do. (Focus group 2)

However, other students experienced that passive facilitation allowed them to test their own mastery. Ahead of the simulated scenarios they were assured that they could ask for guidance if needed. This provided the students with a sense of control. Another way they could “take control” in the situation was to request “time out”. Pausing the scenario allowed students to seek guidance just when needed, which helped them feel less overwhelmed: “We could take ‘time out’ in two of the six scenarios, and it was a welcome opportunity” (Focus group 3).

Students addressed the importance of the facilitator’s flexibility in communicating guidance. Alternating between active and passive guidance to adapt to students’ individual preferences seemed to be a complex relational and pedagogical challenge.

### Emotional influence

In this sub-category, the students addressed how the facilitator’s presence in the simulation room influenced their emotions. They described a variety of feelings, including being motivated, stressed, reassured, or insecure.

The students described three different ways in which the presence of a passive facilitator in the room affected them positively. First, they were able to observe the facilitator’s nonverbal reactions during the simulated scenarios, which helped reassure them that they were doing things right:

I appreciated that the facilitator was always present and observing possible signs of uncertainty about what I should do, and that I was able to see their face to confirm that they recognized that I needed help—and were not sitting behind that window and perhaps thinking, “Oh my gosh, she is not managing the task”. Unless you can see the facilitator’s face, it is easy to have such thoughts. (Focus group 5)

Second, the students reported feeling reassured knowing that a facilitator was present and was focused on their learning outcomes. One student expressed, “I think it is very good that the facilitators are sitting there watching us. I don´t feel performance anxiety” (Focus group 5). Third, the facilitator’s presence could also encourage eager students to manage the situation as well as possible: “The facilitator has a motivational effect on the learning process. In addition, I think you push yourself a little extra when there is a facilitator present” (Focus group 4).

However, students also reported feeling distressed when a facilitator was present in the room. Some students would have preferred to have the facilitator in another room physically separated from the simulation room: “It was distracting to have the facilitator in the room watching. I would have liked to have tried myself, and therefore it would have been better if they were standing in the room next door” (Focus group 2).

For other students, scenarios conducted with a passive facilitator evoked feelings of being left to themselves: “No, I did not get much [support] from my facilitator, and I felt very alone in the situation” (Focus group 4). However, when students were engrossed in a scenario, they sometimes were not aware of the facilitator´s presence in the room:

I was terribly stressed in the situation and not aware of the facilitator being in the room. She might even have been sitting behind me. Anyway, I did not notice if she registered my mistakes because I was totally engrossed in the simulation. (Focus group 5)

The presence of a facilitator in the simulation room evoked a variety of feelings for students, who reported that these emotions influenced their ability to master the simulation.

## Discussion

Overall, the results show that the students thought facilitators played an important role in contributing to their problem-solving during simulation. The results revealed diverse student learning needs, which makes the facilitator’s role more complex in terms of assessing the timing of interventions and providing active and passive support in accordance with students’ individual needs.

For various reasons, students wanted the facilitator nearby during the simulated scenarios. Some students wanted practical support quickly and not waste time if they got stuck when problem-solving. Other students needed verbal or nonverbal guidance to be sure they were on the right track. Still others reported feeling insecure and wanting to have the facilitator in the room in case they needed assistance, what Paige and Morin [23] call the “Stand by me” learning preferences. These students wanted a facilitator to be present in the room and to interrupt them and guide them during the situation so they could learn. According to Vygotsky, a learner might reach a higher order of cognitive learning in an interplay between the environment and guidance from a skilled person [14, 17]. In a collaborative process, the facilitator might use different ways of cuing students to stimulate them to reach their ZPD [33]. The facilitator needs to be aware of when it is appropriate to gradually decrease guidance according to the learner’s learning process [33]. Escher et al. [22] found that facilitators helped participants problem-solve when they were in the same room and could observe details and promptly respond to students’ actions. The ability to provide accurate, specific, and timely feedback is vital to best practice facilitation [18]. In the present study, alternating between passive and active facilitation seemed to support students’ progress in problem-solving and was probably more effective than not providing any cues. This is consistent with cognitive load theory, which holds that learning is maximized when challenges correspond to the learners' level of experience [21].

Students in the present study also revealed a preference for tackling issues on their own, before getting cues from the facilitator. Some students expressed the need to be independent and confident in their own decision-making. Others noted the importance of being able to decide when to ask for help, and still others were inspired by the mere presence of a facilitator in the room. Previous research has described a similar learning perspective as “Let me show you”, which refers to when students want minimal cues from facilitators while problem-solving on their own. Being able to stay focused is important for students’ individual internal learning process and to reach their developmental potential [14, 17]. In simulation, it is advisable to set a time limit for each section to prevent learners from becoming “stranded” [1]. Simulated scenarios ought to be challenging yet manageable for the learner [21]. Therefore, the timing of alternation between being active and passive facilitation is vital to maintain flow of the simulation, and facilitators must be able to assess when to make this switch [12, 21].

Students reported being emotionally affected when the facilitator was too active and interrupted them during the simulated scenarios, or sometimes just by their presence in the room, which made them anxious or disturbed their problem-solving process. A similar learning perspective has been characterized as “Let me think it through”, which includes a preference for not being interrupted by assistance with equipment or redirected by cuing [23]. Such learners were afraid of losing their train of thought and feeling stupid and unable to master the task [23]. Students often report feeling stressed and afraid of making mistakes in situations where others are watching them perform [3, 20]. In SBL, it is essential that facilitators create a trusting environment for students, with dynamic interaction and collaboration, to foster a learner-centered perspective [34] and prevent cognitive overstimulation [21]. Previous research has shown that students must feel psychologically safe to achieve their learning outcomes [20, 35]. Findings in previous research have shown that students must feel psychologically safe to achieve their learning outcomes [20, 35]. Furthermore, the students did not feel they had the support they needed from a facilitator and therefore experienced a feeling of loneliness.

The results also show that some students did not feel they had the support they needed from a facilitator and therefore experienced a feeling of loneliness. These students may have felt defeated and disappointed by the facilitator’s passivity. Paige and Morin [23] identify a similar learning perspective, “The agony of defeat”, which applies to students who wanted to be interrupted during the simulated scenario to avoid the feeling of defeat and to leave the simulation with good emotions. Dieckmann and Ringsted [16] recommend that learners be allowed to ask for more time, or a clearer presentation of the scenario when confronted with scenarios that are too difficult. However, students might find it hard to ask for this. Madsgaard and Røykenes [20] reported that facilitators simplified complex scenarios if they observed that students were uncomfortable or unsatisfied during a simulation. According to Jeffries et al. [12] and cognitive load theory [21], a level of complexity that is too high is unfavourable to learning. Kelly et al. [8] emphasized that timing cues adapted to each student is challenging for facilitators but an important skill to focus on.

The experience of not knowing what to do in a critical situation was momentous for some of the students, who reflected on the consequences of such unpreparedness in real life. Situations like this could be viewed in the light of what Kelly et al. [8] describe as a failure not to bridge the gap between simulation and clinical practice. SBL aims to prepare students for unforeseen events [7, 18]. To this end, simulation design ought to include pedagogical factors such as familiarity, warning and escalation, and cues that can help students master the situation [9]. Facilitators must be aware of whether students are responding to warning signs and then actively support and guide them in the right direction [20]. In SBL, it is often emphasized that a sense of mastery promotes learning [36]. However, students’ learning needs vary a lot [23]. Students who feel engaged and not defeated might achieve greater competence. Paige and Morin [23] describe a similar learning perspective as, “I’m engaging and so should you”, in which students experienced the simulation as though it were real, as a wake-up call, and did not feel defeated. Likewise, for passive students, such an experience might be just what is needed to stimulate their attention and get them to “engage fully, think more deeply, and learn for mastery” [37].

### Limitations

A purposive sample with nursing students from one university attending the same SBL program was used. Therefore, the results might be somewhat one-sided compared with other nursing studies from other universities. The results were not reviewed with the students as it was difficult to gather them together due to other curriculum demands.

## Conclusions

The main category “alternating between active and passive facilitation” portrays how facilitators in simulated scenarios need to be skilled in providing individually suitable cues at the right time to students with a variety of learning preferences. Facilitators must have relational, pedagogical, and emotional competence in addition to clinical, technical, and SBL skills to adjust to students’ different learning needs and adapt to their preference for either active or passive cues. This study has shown that facilitator education should emphasize the importance of knowing when to remain passive and when to intervene. Because of the challenging and complicated nature of the role, more research is needed to explore how facilitators should adjust cues individually to promote effective SBL in nursing education.

## Supplementary Information

**Additional file 1.** Setting.

**Additional file 2.** Interview guide.

## Data Availability

The datasets generated and/or analyzed during the present study are not publicly available due to the limited sample and the importance of preserving the anonymity of the participants but are available from the corresponding author on reasonable request.

## Abbreviations

HFS: High-fidelity simulation
SBL: Simulation-based learning
