# Supplementary material for: Alternating between active and passive facilitator roles in simulated scenarios: a qualitative study of nursing students’ perceptions
Source: Adv Simul (Lond). 2022 Oct 29;7:37. doi: 10.1186/s41077-022-00233-0 (PMC9618220; doi:10.1186/s41077-022-00233-0)
Supplement: Supplementary file 1 — Additional file 1. Setting. [file 41077_2022_233_MOESM1_ESM.docx]

# Additional file 1.

**Organization of the six scenarios:**
**Prebriefing:** 15 minutes to demonstrate the manikin. The students are informed about the role of operator and facilitator; the operator sits in a room with a one-way window facing the room where the scenario runs. The operator manages and assumes the voice of the simulator. The facilitator is present in the room where the simulation takes place; they remain in the background but can provide small input or cues if the scenario is about to stop. In some of the scenarios, students can request a "time-out”.

**Briefing:** 15 minutes. The students are informed about the duty of confidentiality and how long the various elements of the simulation last. Scenario-specific equipment and conditions are informed about, and emphasis is placed on creating a safe atmosphere during the whole scenario.

**Simulated scenario**: 15 minutes. After the simulated scenario, the students acting as nurses are watching the video recordings, and the observers and facilitator are planning their feedback: 15 minutes.
**Debriefing:** Alle students gather to a facilitator led debriefing for 50 minutes.

|  | **1. Angina pectoris** | **2. Hypoglycemia** | **3. Cardiac arrest** |
| --- | --- | --- | --- |
| Learning outcomes | - Observes, assesses, and prioritizes action  - Communicates efficiently  - Takes the lead | - Observes, assesses, and prioritizes action  - Communicates efficiently  - Takes the lead | - Identifies signs of cardiac arrest. - Performs CPR according to current   guidelines - Retrieves and prepares the necessary   equipment - Communicates effectively |
| Role description | Two students act as nurses, the remaining students as observers. | Two students act as nurses, one student is a friend who follows the patient, and the remaining students act as observers. | Three students act as nurses, the remaining students as observers. |
| Utilities | The operator acts as a doctor who can be called. | The operator acts as a doctor who can be called and plays next of kin who call the hospital. | The operator acts as a doctor who can be called. |
| Description of the patient’s health condition | Carl Jensen, male, 56 years old. Weight 95 kg, height 175 cm. Admitted to the emergency room due to sudden onset of chest pain and wheezing.  When the scenario begins:  The patient is awake and ready, seems scared and anxious, sweat beads on the forehead, breathes quickly.  The patient is seen by the doctor, and he ordinates glyceryloltrinitrat sublingually and acetylsalicylic acid. After the second administration of glyceryloltrinitrat, the patient suffers a drop in blood pressure. | Susan Hansen, female, 18 years (age majority). Weight 60 kg, height 170cm. Admitted to the ward with type 1 diabetes.  For the past three months, she has been experienced increasing thirst, frequent urination and weight loss, and has generally felt unwell. She has no signs of infection, no known allergies, and no fever during her stay.  On the actual day, the patient was walking in the park with a friend. The friend explained that Ms. Hansen suddenly became irritable, she expressed hunger, felt slightly shaky and light-headed. The friend became concerned and escorted her to the ward.  When the scenario begins:  The patient still seems somewhat unconcentrated and is uncooperative.  At the end of the scenario, her mother calls the hospital and asks for information about the patient's condition. The patient has informed the nurses that she does not want the mother to get any information about her status. | Carl Jensen, male, 56 years. Admitted to the medical ward 24 hours after he visited the emergency room on his own due to his symptoms (murmuring chest pains and wheezing).  Now he has suffered new severe chest pains, no effect of glyceryloltrinitrat. He has been given a total of Morphine 15 mg intravenous, blood tests have been taken and electrocardiogram shows ST-elevation consistent with acute heart attack. He is waiting for a transfer to another hospital to perform percutaneous coronary intervention.  When the scenario begins:  The patient is still in pain, and a new dose of Morphine 5mg intravenous must be set. In a short period of time, he will have a cardiac arrest. |
| Expected progress | The students are expected to follow the local facility protocol for the treatment of chest pain suggestive of ischemia. | The students are expected to recognize symptoms of hypoglycemia, treat appropriately, and keep patient information confidential.  During the phone call with the mother, the students are expected to provide duty of confidentiality. | The students are expected to follow the local facility protocol for the treatment of cardiac arrest. |
|  | **4. Postoperative bleeding** | **5. Worsening of obstructive lung disease** | **6. Ileus** |
| Learning outcome | - Observes, assesses, and prioritizes action  - Communicates efficiently  - Takes the lead | - Observes, assesses, and prioritizes action  - Communicates efficiently  - Takes the lead. | - Observes, assesses, and prioritizes action  - Communicates efficiently  - Takes the lead |
| Role description | Two students act as nurses, the remaining students as observers. | Two students act as nurses, the remaining students as observers.  One of the students acts as next of kin. | Two students act as nurses, the remaining students as observers. |
| Utilities | The operator acts as a doctor who can be called. | The operator acts as a doctor who can be called. | The operator acts as a doctor who can be called. |
| Description and development of the situation | Lars Berntsen, male, 76 years old. He came to the emergency room last night. He had fallen outdoors and incurred a fractura colli femoris on the left side. He underwent immediate surgery, and a hip replacement was inserted.  When the scenario begins:  12 o’clock: First postoperative day. The patient calls and complains that he is dizzy, in pain and feeling very tired. He seems alert and adequate, has a pale complexion, vomits, has rapid breathing, and there is blood in the bandage. His blood pressure drops and he loses consciousness. | Vidar Broby, male, 69 years old. Weight 70 kg, height 172 cm.  In the emergency department due to worsening chronic obstructive pulmonary disease, COPD, stage 3. He complained of increasing fatigue and difficulty sleeping at night. He has been increasingly suffering from mucus production and coughing in recent days.  His wife is with him, she's worried about the situation. The patient is awake and alert, uses respiratory auxiliary muscles, has a barrel-shaped chest and "drumstick fingers". The patient coughs something and brings up some clear expectorate. He sleeps with O^2^ 1 l/min and uses nebulizer.  When the scenario begins:  The patient condition has deteriorated, his SpO^2^ has fallen below his habitual level. The patient is in distress. | Stian Carlsen, male, 52 years old. Weight 76 kg, height 160 cm.  Has previously undergone several surgical procedures; cholecystectomy, appendectomy, right-sided inguinal hernia. Alle performed more than 5 years ago.  He arrives at the emergency room due to severe abdominal pain and vomiting in recent days. The stomach is bloated. He has dry mucous membranes. The urine has been concentrated the recent days, with no urine output in the last 24 hours. He's been feeling dizzy and tired all afternoon and has eaten and drunk little during the last few days.  Upon admission, the patient is lethargic and pale, expressed pain.  When the scenario begins:  The patient is becoming increasingly dizzy and tired. |
| Expected progress | The students are expected to demonstrate basic assessment skills to detect signs and symptoms of postoperative bleeding and provide appropriate treatment. | The students are expected to initiate a respiratory assessment and provide appropriate interventions. | The students are expected to demonstrate basic assessment to detect signs and symptoms of severe dehydration and impending hypovolemic shock. They are expected to provide appropriate treatment. |

The scenarios were inspired by existing scenarios created by the National League for Nursing and Laerdal. They were refined in collaboration with practicing nurses to suit a Norwegian context.
Laerdal Medical Corporation 2008: Simulation in Nursing Education, VitalSim Scenarios, SP7202 rev A.
