# Supplementary material for: Alternating between active and passive facilitator roles in simulated scenarios: a qualitative study of nursing students’ perceptions
Source: Adv Simul (Lond). 2022 Oct 29;7:37. doi: 10.1186/s41077-022-00233-0 (PMC9618220; doi:10.1186/s41077-022-00233-0)
Supplement: Supplementary file 2 — Additional file 2. Interview guide. [file 41077_2022_233_MOESM2_ESM.docx]

**Additional file 2: Interview guide**

| **Interview guide**  We want to ask you some question about how you perceive the importance of the facilitator’s role in the simulated scenarios in simulation-based learning   - How did you experience the importance of the facilitator’s role in the simulated scenarios when you were executing the simulation activity? - How did you experience the facilitator’s way of providing you with actual cues according to your needs during the simulation in relation to:   1) the scenario?  2) medical equipment in the simulation room?  3) the manikin?   - Did you have the opportunity to pose questions during the simulated scenario? In what way did the facilitator respond to your questions adequately? |
| --- |
